# Quantification of 11 enzyme activities of lysosomal storage disorders using liquid chromatography-tandem mass spectrometry

**DOI:** 10.1016/j.ymgmr.2018.08.005

**Published:** 2018-09-07

**Authors:** Mari Ohira, Torayuki Okuyama, Ryuichi Mashima

**Affiliations:** Department of Clinical Laboratory Medicine, National Center for Child Health and Development, 2-10-1 Okura, Setagaya-ku, Tokyo 157-8535, Japan

## Abstract

Lysosomal storage disorders (LSDs) are characterized by the accumulation of lipids, glycolipids, oligosaccharides, mucopolysaccharides, and other biological substances because of the pathogenic deficiency of lysosomal enzymes. Such diseases are rare; thus, a multiplex assay for these disorders is effective for the identification of affected individuals during the presymptomatic period. Previous studies have demonstrated that such assays can be performed using liquid chromatography-tandem mass spectrometry (LC-MS/MS) with multiple reaction monitoring (MRM) detection. An assay procedure to quantify the activity of 11 enzymes associated with LSDs was provided. First, a validation study was performed using dried blood spot (DBS) samples with 100% and 5% enzyme activity for quality control (QC). Under the assay condition, the analytical range, defined as the ratio of the peak area of the enzyme reaction products from the DBS for QC with 100% enzyme activity to that from the filter paper blank sample, was between 14 for GALN and 4561 for GLA. Based on these results, the distribution of the enzyme activity for the 11 LSD enzymes was further examined. Consistent with the previous data, the enzyme activity exhibited a bell-shaped distribution with a single peak. The averaged enzyme activity for the healthy neonates was as follows: GLA, 3.80 ± 1.6; GAA, 10.6 ± 4.8; IDUA, 6.4 ± 2.3; ABG, 8.6 ± 3.1; ASM, 3.3 ± 1.1; GALC, 2.8 ± 1.3; ID2S, 16.7 ± 6.1; GALN, 1.2 ± 0.5; ARSB, 17.0 ± 8.7; NAGLU, 4.6 ± 1.5; and GUSB, 46.6 ± 19.0 μmol/h/L (mean ± SD, n = 200). In contrast, the enzyme activity in disease-affected individuals was lower than the minimum enzyme activity in healthy neonates. The results demonstrate that the population of disease-affected individuals was distinguished from that of healthy individuals by the use of LC-MS/MS.

## 1 Introduction

Lysosomal storage disorders (LSDs) are characterized by the accumulation of biological substances, such as glycolipids, lipids, oligosaccharides, and mucopolysaccharides, in the lysosomes as a result of the pathogenic defects of the lysosomal enzymes [1,2]. The accumulating evidence suggests that the efficacy of existing therapies for the treatment of LSDs is related to newborn screening [3,4]. The best known example includes a study of Pompe disease in Taiwan, which showed that the survival rate was significantly improved without ventilator use [5]. In this case, the treatment was initiated during the presymptomatic period immediately after newborn screening. Among the currently available therapies, enzyme replacement therapy (ERT) has been widely accepted as an effective treatment option. It is known that several LSDs, including Pompe disease; Fabry disease; mucopolysaccharidosis (MPS) I, II, IVA, and VI; and Gaucher disease can be treated with ERT [2].

Mass spectrometry (MS)-based LSD assays were first reported by Dr. M.H. Gelb at the University of Washington [6,7]. The principle of this method is the inclusion of an internal standard (IS) for each assay to quantify the accumulation of enzyme reaction products using tandem mass spectrometry (MS/MS) with multiple reaction monitoring (MRM). The IS for each enzyme reaction was synthesized using deuterium-labeled compounds, leading to the higher accuracy of the quantitative results [8]. Based on this, this technique has been significantly improved over the more than one decade that it has been studied. First, the substrates for enzyme assay are synthetic thus non-endogenously existing, leading to the accurate quantification of elevating enzyme reaction products with minimal background. Secondly, this MRM-based assay can perform several assay in a single buffer while each of these assay needs to be performed individually using fluorescence-active substrate [9]. Thirdly, to increase the number of enzymes, an initial study established an assay for 5-plex LSD enzymes, including α-glucosidase (GAA) for Pompe disease, α-galactosidase A (GLA) for Fabry disease, acid β-glucosidase (ABG) for Gaucher disease, acid sphingomyelinase (ASM) for Niemann-Pick disease type A/B, and galactosylcerebrosidase (GALC) for Krabbe disease [7], followed by the inclusion of α-L-iduronidase (IDUA) for mucopolysaccharidosis (MPS) I in a subsequent study [10]. Finally, to accommodate the large number of DBS samples under current NBS platform, the assay was performed using flow-injection analysis under high-throughput assay conditions (8). Based on these innovations, these reagents were used for several pilot studies reported in Austria [11], Taiwan [12], and the United States (U.S.) [10]. Based on these studies, NBS for LSD has been implemented in Taiwan [12] and some state in the U.S. [13].

Because the number of new therapies has been emerging based on new technologies, the number of assay reagents for LSD enzyme activity has also been growing. Assay reagents for iduronate-2-sulfatase (ID2S) for MPS II, *N*-acetylgalactosamine-6-sulfatase (ARSB) for MPS IVA, *N*-acetylgalactosamine-4-sulfatase (GALN) for MPS VI, *N*-acetyl-α-D-glucosaminidase (NAGLU) for MPS IIIB, and β-glucuronidase (GUSB) for MPS VII [14], as well as lysosomal acid lipase and biotinidase for the associated deficiencies [15,16] were developed in previous studies. These enzyme activities can be quantified with a combination of enzymes. For example, a previous study reported that the enzyme activities for the above-mentioned 6 LSDs were quantified with ID2S, ARSB, and GALN using liquid chromatography-tandem mass spectrometry (LC-MS/MS) [17]. Subsequently, the same group developed another 6-plex assay for ID2S, ARSB, GALN, NAGLU, GUSB, and tripeptidyl peptidase 1 [14]. Previous studies have reported that enzyme assays for ID2S, ARSB, GALN, NAGLU, GUSB were performed using LC-MS/MS [14,17]. One reason for this choice is the accumulation of artifactually produced heat-labile compounds from the enzyme substrate during MS detection [14,17]. For the accurate quantification of the enzyme activity, these peaks should be minimized either by chromatographic separation or optimizing MS detection. Although this assay lasts 2 min, this method can be used for NBS. The assay time in this study was close to that in the recently published assay procedure that targets NBS [14,16,17]. A chromatographic condition that enables the quantification of enzyme activity for ID2S, ARSB, and GALN aiming for high throughput quantification was reported in a previous study [18]. The current study has extended that previous study on the multiplex quantification of LSD enzymes involving GAA, GLA, IDUA, ABG, ASM, GALC, NAGLU, and GUSB by using LC-MS/MS.

## 2 Experimental procedures

### 2.1 Materials

The reagents required for the 6-plex LSD enzyme assay for the GAA, GLA, IDUA, ABG, ASM, and GALC were purchased from PerkinElmer (Waltham, MA). The reagents for the ID2S, GALN, and ARSB were purchased from PerkinElmer under a custom manufacturing agreement. The reagents for the NAGLU and GUSB were provided by Prof. Michael H. Gelb (University of Washington, WA). The *N*-Acetylglucosamine thiazoline (NAG-thiazoline) was purchased from Toronto Research Chemicals (Toronto, ON, Canada). The acetonitrile was purchased from Thermo Fischer Scientific (Tokyo, Japan). The deionized water was obtained from a Milli-Q water system (Millipore, Milford, MA). The formic acid was purchased from Kanto Chemical (Tokyo, Japan). A set of DBS samples with high (100%) and low (5%) activity was provided by PerkinElmer for QC. The other reagents used in this study were of the highest commercially available grade.

### 2.2 Approval by institutional research ethics board

This study was approved by the Research Ethics Board of the National Center for Child Health and Development.

### 2.3 DBS

The DBS samples were stored at −20 °C according to previous procedures [19]. One untreated MPS I-affected individual, four MPS II-affected individuals with ERT, one untreated MPS IIIB-affected individual, two untreated MPS IVA-affected individuals, and two MPS VI-affected individuals with ERT were examined. We used newborn specimens for all controls.

### 2.4 Reaction-for GAA, GLA, IDUA, ABG, ASM, and GALC (Plate A)

The assay cocktail contained the known concentrations of substrate for the GAA for Pompe disease (0.35 mM), the GLA for Fabry disease (1.2 mM), the IDUA for MPS I (0.25 mM), the ABG for Gaucher disease (0.5 mM), the ASM for Niemann-Pick disease type A/B (0.75 mM), and the GALC for Krabbe disease (0.85 mM) and of IS for the GAA (24 μM), GLA (24 μM), IDUA (15 μM), ABG (20 μM), ASM (15 μM), and GALC (10 μM) [20,21]. All of the assays were carried out with a 3-mm punch in 30 μL of assay cocktail in a polypropylene 96-well plate (#260252, Thermo Fisher Scientific, Tokyo) and incubated at 37 °C for 20 h. To terminate this enzyme reaction, a mixture of methanol/ethyl acetate (50/50, 100 μL) was added. Next, to extract the enzyme reaction products, ethyl acetate (400 μL) and 0.5 M sodium chloride (200 μL) were added and mixed vigorously using a pipette. After the centrifugation of these plates at 700 ×*g* for 5 min at room temperature using a plate centrifuge (model PlateSpinII, Kubota, Tokyo, Japan), an aliquot of the organic layer (200 μL) was transferred to a fresh 96-well plate.

### 2.5 Reaction for ID2S, ARSB, GALN (Plate B)

The assay cocktail contained the known concentrations of substrate for the ID2S for MPS II (0.5 mM), the GALN for MPS IVA (1 mM), and the ARSB for MPS VI (1 mM) and of IS for the ID2S (5 μM), GALN (5 μM), and ARSB (5 μM) [18]. The enzyme reactions were performed in 50 mM ammonium acetate (pH 5.0) containing 7.5 mM barium acetate, 5 mM cerium acetate, and 2 mM (*Z*)-Pugnac. All of the assays were carried out with a 3-mm punch in 30 μL of the assay cocktail in a polypropylene 96-well plate and incubated at 37 °C for 20 h. To terminate this enzyme reaction, a mixture of methanol/ ethyl acetate (50/50, 100 μL) was added. Next, to extract the enzyme reaction products, ethyl acetate (400 μL) and 0.5 M sodium chloride (200 μL) were added and mixed vigorously using a pipette. After the centrifugation of these plates at 700 ×*g* for 5 min at room temperature using a plate centrifuge, an aliquot of the organic layer (200 μL) was transferred to a fresh 96-well plate.

### 2.6 Reaction for NAGLU and GUSB (Plate C)

The assay cocktail contained the known concentrations of substrate for the NAGLU for MPS IIIB (0.5 mM) and the GUSB for MPS VII (0.5 mM) and of IS for the NAGLU (10 μM) and GUSB (10 μM) [14]. The enzyme reactions were performed in 50 mM ammonium acetate (pH 5.0) containing 7.5 mM barium acetate, 5 mM cerium acetate, and 0.1 mM NAG-thiazoline (Toronto Research Chemicals, Ontario, Canada) [14]. All of the assays were carried out with a 3-mm punch in 30 μL of assay cocktail in a polypropylene 96-well plate (#260252, Thermo Fisher Scientific, Tokyo) and incubated at 37 °C for 20 h. To terminate this reaction mixture, a mixture of methanol/ethyl acetate (50/50, 100 μL) was added. Next, to extract the enzyme reaction products, ethyl acetate (400 μL) and 0.5 M sodium chloride (200 μL) were added and mixed vigorously using a pipette. After the centrifugation of these plates at 700 ×*g* for 5 min at room temperature using a plate centrifuge, an aliquot of the organic layer (200 μL) was transferred to a fresh 96-well plate.

### 2.7 Reconstitution of enzyme reaction products for 11-plex complete assay and 6/3/2-plex separate assay

For the 11-plex assay, an aliquot of the ethyl acetate layer from Plates A (200 μL), B (200 μL), and C (200 μL) were combined in a deep-well plate. Next, the solvent of the extracts was removed under a nitrogen stream. Finally, the enzyme reaction products were reconstituted with a reconstitution solvent (acetonitrile/water/formic acid = 80/20/0.1, 300 μL). In this study, it was estimated that a 3-mm DBS punch contained 3.1 μL of whole blood. Enzyme activity was calculated in μmol/h/L blood as was previously reported [22].

### 2.8 LC-MS/MS

An aliquot (5 μL) was injected into a Quattro Premier XE tandem mass spectrometer equipped with an Acquity UPLC chromatograph (Waters). An aliquot of the samples was injected by autosampler into an analytical column ACQUITY CSH C18 (2.1 mm inner diameter × 30 mm length, 1.7 μm particle size, Waters) equilibrated with 80% mobile phase A (0.2% formic acid in 5% acetonitrile/95% water) and 20% mobile phase B (0.2% formic acid in acetonitrile) at a flow rate of 0.6 mL/min at 40 °C. The enzyme reaction product and IS were eluted with 20% mobile phase B for 0–0.1 min, 20–100% for 0.1–1.0 min, 100% for 1.0–1.5 min, and 20% for 1.51–2.0 min. The data were collected using MassLynx software V4.1 (Waters). The other analytical conditions are described in Supplementary Tables 1–3.

## 3 Results

### 3.1 Assay validation

**Fig. 1:**
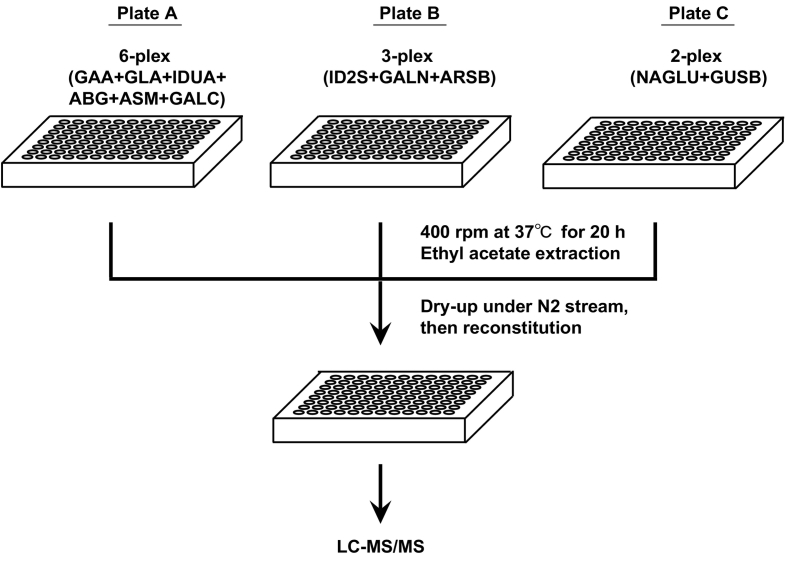
Outline of assay procedure for 11-plex LSD assay using LC-MS/MS. Plate A was used for 6-plex assay for GAA, GLA, IDUA, ABG, ASM, and GALC. Plate B was used for 3-plex assay for ID2S, GALN, and ARSB. Plate C was used for 2-plex assay for NAGLU and GUSB. For 11-plex assay, an aliquot of ethyl acetate layer from Plate A (200 μL), Plate B (200 μL), and Plate C (200 μL) were combined followed by evaporation of solvent under nitrogen stream. For comparison, we performed 6/3/2-plex assay using Plate A/B/C. In this case, an aliquot of ethyl acetate layer was collected in separate 96-well deep plates. Following the evaporation of solvent under nitrogen stream, reaction products were solubilized with Reconstitution solvent (acetonitrile/water/formic acid = 80/20/0.1, 300 μL).

**Fig. 2:**
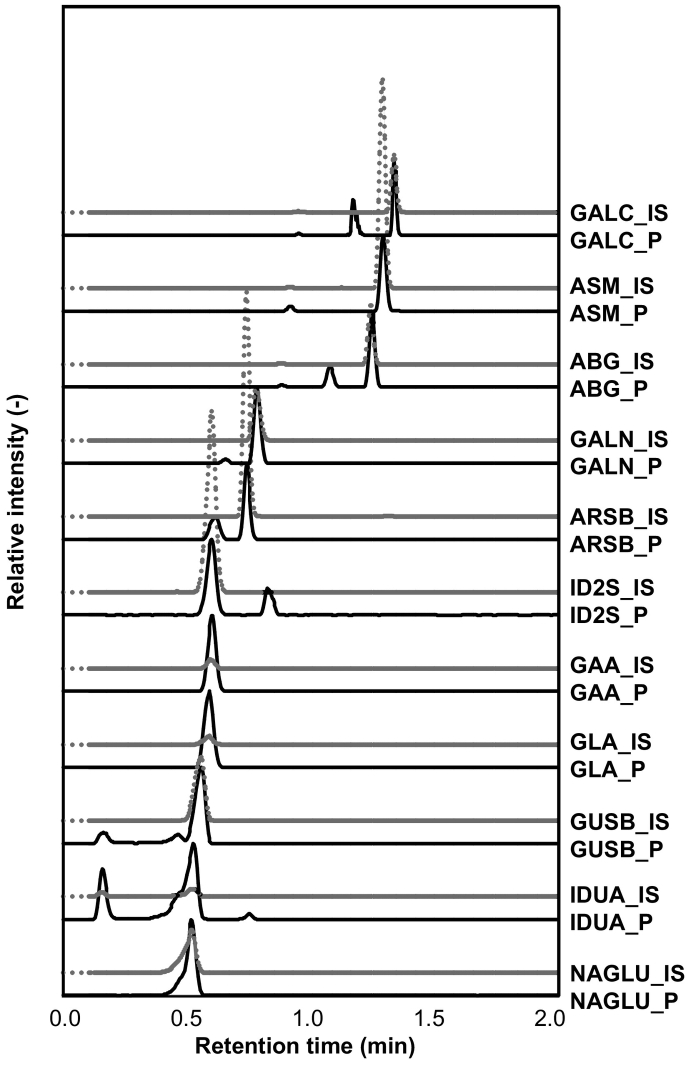
A representative chromatogram obtained from a healthy control newborn. Y-axis represents relative intensity normalized to the enzyme reaction product as 100%. For the enzyme reaction, a 3-mm punch of DBS was reacted as described in Experimental Procedure at 37 °C for 20 h. Enzyme reaction products were extracted into ethyl acetate, dried under nitrogen stream and solubilized with Reconstitution solvent (300 μL) and injected onto UPLC-MS/MS (5 μL). Black line represents the chromatogram for the product of enzyme reaction and gray line represents that for IS.

A chromatographic condition for ID2S, ARSB, and GALN aiming for a high throughput assay was previously reported [18]. This assay method was extended for quantifying 11 LSD enzyme activities by using LC-MS/MS (Fig. 1). An improved chromatographic condition for IS and enzyme reaction products for 11 LSD enzymes were developed based on the preliminary experiments (Fig. 2). During the course of the current study, it was observed that the enzyme activity for the NAGLU was always attenuated when this reaction was performed with the ID2S, ARSB, and GALN assays (data not shown). It was found subsequently that (*Z*)-Pugnac, an inhibitor for O-linked *N*-acetylglucosamine, was included in this batch of ID2S, ARSB, and GALN reagents at the time of production (personal communication from the manufacture). This implies that the suppression for NAGLU observed above in the preliminary 11-plex assay could be associated with (*Z*)-Pugnac in assay reagent for ID2S, ARSB, and GALN. Thus, a decision was made to examine a 11-plex assay using a combination of three 96-well plates (6/3/2-plex assay using Plate A/B/C). For comparison, a 6-plex assay for the GAA, GLA, IDUA, ABG, ASM, GALC; a 3-plex assay for the ID2S, ARSB, and GALN; and a 2-plex for the NAGLU and GUSB were performed (Fig. 1). Under the assay conditions in this study, the effect of the ion suppression was not obvious because the peak areas for the IS in the 11-plex LSD enzyme assay were not as severely attenuated as those in the 6/3/2-plex assay (Supplementary Fig. 1).

Next, an assay validation study using QC DBS with a known percentage of leukocytes was performed. These QC DBSs contained 100% and 5% of enzyme activity of whole blood (See Experimental Procedure). As was previously demonstrated, the accumulation of enzyme reaction products was proportional to the percentage of enzyme activity in the DBS (Supplementary Fig. 2). As was demonstrated, the CV values for the QC DBS with high enzyme activity for intraday and interday precision were within 12% in the 11-plex assay (Table 1). The analytical range was defined as the ratio of peak areas from the enzyme reaction product from the QC DBS with 100% enzyme activity to those from the filter paper blank samples [8,22]. In the 11-plex assay, the value of analytical range for each enzyme was as follows: GLA, 4561; GAA, 4107; IDUA, 210; ABG, 213; ASM, 309; GALC, 238; ID2S, 156; GALN, 14; ARSB, 102; NAGLU, 201; and GUSB, 504 (Table 2). These results appeared to be similar to the values for the analytical ranges obtained from the 6/3/2-plex assay (Table 2). It must be noted that 0.1 mM NAG-thiazoline, an inhibitor for hexosaminidase A, was always present in the assay cocktail for the NAGLU and GUSB, otherwise the enzyme activity for the NAGLU was suppressed because of the endogenous hexosaminidase A activity (data not shown).

**Table 1:** Interday and intraday CV values for 11 LSD enzyme activities using LC-MS/MS.

	Sample	Assay procedure	GLA	GAA	IDUA	ABG	ASM	GALC	ID2S	GALN	ARSB	NAGLU	GUSB	n
			CV (%)											
Intraday	Low	11-plex	7	7	7	8	17	19	20	14	8	9	7	7
	Low	6/3/2-plex	12	7	4	7	16	6	17	14	9	9	6	7
	High	11-plex	6	3	5	5	12	8	11	5	12	5	4	7
	High	6/3/2-plex	5	5	9	7	13	10	16	15	6	9	5	7
Interday	Low	11-plex	10	13	15	21	22	4	10	10	14	12	9	3
	Low	6/3/2-plex	15	12	2	25	14	11	23	14	20	11	6	3
	High	11-plex	2	8	8	2	4	7	6	2	5	10	11	3
	High	6/3/2-plex	4	7	7	6	15	8	6	3	1	11	2	3

Low, QC DBS with 5% enzyme activity; High, QC DBS with 100% enzyme activity.

**Table 2:** Analytical ranges for 11 LSD enzyme activities by LC-MS/MS.

Assay procedure	Plate name	Enzyme	11-plex assay	6/3/2-plex assay
6-plex	Plate A	GLA	4561	2899
		GAA	4107	4411
		IDUA	210	263
		ABG	213	206
		ASM	309	267
		GALC	238	173
3-plex	Plate B	ID2S	156	279
		GALN	14	15
		ARSB	102	144
2-plex	Plate C	NAGLU	201	214
		GUSB	504	632

### 3.2 Distribution of enzyme activity in neonatal population

**Fig. 3:**
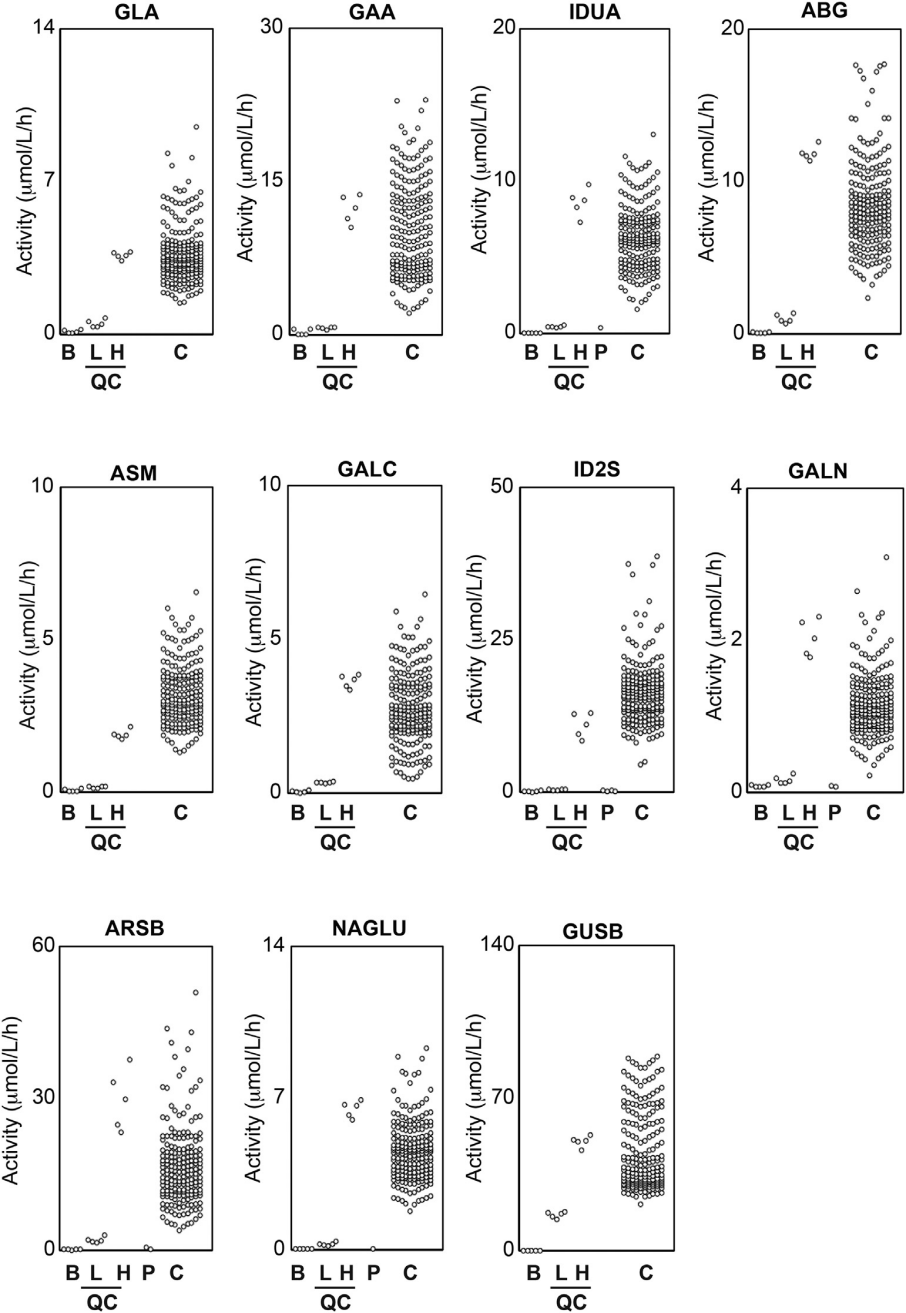
Enzyme activities in DBS of controls and disease-affected individuals examined using LC-MS/MS. The enzyme activities in filter paper blank (B, n = 4), in DBS for QC with 100% enzyme activity (H, n = 4), for QC with 5% enzyme activity (L, n = 4), in DBS of healthy controls (C, n = 200), and of patients (P) with an MPS I-affected individual (n = 1), MPS II-affected individuals (n = 4), an MPS IIIB-affected individual (n = 1), MPS IVA-affected individuals (n = 2), and MPS VI-affected individuals (n = 2) were presented.

The distribution of the enzyme activity in the healthy controls for the 11 LSD enzymes was examined. There was one peak in the distribution of the activity in the 11 LSD enzymes (Supplementary Fig. 3). Note that enzyme activity of neonates is usually higher than that of adults due to higher leukocyte counts: our results were comparable to previous studies because the enzyme activity for neonatal controls were examined (Table 3). Next, the enzyme activity in the disease-affected individuals was examined using this LC-MS/MS-based assay. Generally, these samples showed very low enzyme activity similar to that in the filter paper blank samples (Fig. 3).

**Table 3:** Comparison of enzyme activities for LSDs using MS/MS-based technique.

Investigator		Ohira M	Mashima R	Mashima R	Mashima R	Spacil Z	Liu Y	Elliott S
Reference		This study	[18]	[20]	[21]	[17]	[14]	[8]
Year		2018	2017	2016	2017	2013	2017	2016
Method		LC-MS/MS	LC-MS/MS	LC-MS/MS	FIA-MS/MS^a^	LC-MS/MS	LC-MS/MS	FIA-MS/MS
Run time (min)		2	2	10	1	1.8	2	1
Enzyme activity (μmol/h/L)	GLA	3.8	NR	8.3	2.8	0.82	NR	17.33
	GAA	10.6	NR	24.1	6.6	3.04	NR	12.41
	IDUA	6.4	NR	5.6	2.9	14.05	NR	6.56
	ABG	8.6	NR	13.0	4.6	9.89	NR	12.69
	ASM	3.3	NR	4.5	1.4	1.62	NR	6.03
	GALC	2.8	NR	3.5	2.1	0.48	NR	5.04
	ID2S	16.7	19.6	NR	NR	0.23	16.1	NR
	GALN	1.2	1.7	NR	NR	1.54	0.67	NR
	ARSB	17.0	13.4	NR	NR	1.16	4.4	NR
	NAGLU	4.6	NR	NR	NR	NR	1.6	NR
	GUSB	46.6	NR	NR	NR	NR	28.5	NR
	TPP1	NR	NR	NR	NR	NR	35.9	NR

a Acetonitrile was used as the solvent. NR, not reported.

The activity of the tested LSD enzymes in the healthy controls was as follows (mean ± SD, μmol/h/L, n = 200): GLA enzyme activity, 3.8 ± 1.6 (median, 3.5; min, 1.5; max, 12.5); GAA enzyme activity, 10.6 ± 4.8 (median, 10.0; min, 2.1; max, 26.8); IDUA enzyme activity, 6.4 ± 2.3 (median, 6.2; min, 1.6; max, 14.3); ABG enzyme activity, 8.6 ± 3.1 (median, 8.1; min, 2.4; max, 18.8); ASM enzyme activity, 3.3 ± 1.1 (median, 3.0; min, 1.3; max, 9.3); GALC enzyme activity, 2.8 ± 1.3 (median, 2.6; min, 0.5; max, 8.0); ID2S enzyme activity, 16.7 ± 6.1 (median, 16.0; min, 4.5; max, 47.7); ARSB enzyme activity, 17.0 ± 8.7 (median, 15.5; min, 3.9; max, 55.5); GALN enzyme activity, 1.2 ± 0.5 (median, 1.1; min, 0.2; max, 3.9); NAGLU enzyme activity, 4.6 ± 1.5 (median, 4.5; min, 1.8; max, 10.7); and GUSB enzyme activity, 46.6 ± 19.0 (median, 39.0; min, 21.3; max, 124.1).

In addition, the enzyme activity in the disease-affected individuals was assessed. The IDUA enzyme activity found for one MPS I-affected individual was 0.39 μmol/h/L, the mean ID2S enzyme activity for four MPS II-affected individuals was 0.18 ± 0.10 μmol/h/L (mean ± SD, median, 0.18; min, 0.09; max, 0.30), the NAGLU enzyme activity for one MPS IIIB-affected individual was 0.05 μmol/h/L, the mean GALN enzyme activity for two MPS IVA-affected individuals was 0.08 μmol/h/L (min, 0.07; max, 0.09), and the mean ARSB enzyme activity for two MPS VI-affected individuals was 0.43 μmol/h/L (min, 0.21; max, 0.65).

## 4 Discussion

This study has demonstrated a quantification procedure for 11-plex LSD enzyme activity using LC-MS/MS. First, the assay performed 3 separate reactions at 37 °C for 20 h. The reaction products were then combined in a single plate, and the enzyme activity was subsequently determined through each accumulating enzyme reaction product by LC-MS/MS (Fig. 1). The enzyme activity in the QC DBS with 100% and 5% enzyme activity of whole blood seemed proportional to the known concentration of active enzymes (Supplementary Fig. 2). In this investigation, the intraday and interday CV values for the QC DBS with high enzyme activity were within 12 and 11% (Table 1). The averaged enzyme activity for the 11 LSD enzymes was similar to those reported values (Table 3). Although the examples were still limited, the enzyme activity in the disease-affected individuals was generally close to that in the filter paper blank samples (Fig. 3). It is important to note that the choice of an inhibitor depends on an assay of which enzyme activity to be measured. A previous study carefully selected NAG-thiazoline as a single inhibitor to perform multiplex assay for ID2S, ARSB, GALN, NAGLU, GUSB, and TPP1 [14].

NBS for LSDs using an MS/MS-based technique has been implemented in several states in the U.S. and Taiwan [3,12]. Currently, the selected disorders for NBS have one or more established therapies. The effectiveness of NBS for LSDs was best presented in a study on Pompe disease in Taiwan, which showed that the detection of affected individuals by NBS during the presymptomatic period leads to the prompt initiation of treatment, leading to better therapeutic outcomes [5]. It is well known that the distribution of LSDs depends heavily on the genetic background and/or geographic location. For example, a subtype of MPSs has a unique geographic distribution. MPS I, a prototypical MPS caused by IDUA, is found mostly in Caucasian populations [23], and MPS II, a distinct type of MPS caused by ID2S exhibiting an accumulation of heparin sulfate and dermatan sulfate similar to that in MPS I, is predominant in Asian populations [24,25]. In addition, a high prevalence of MPS VI has been observed in some of the Brazilian population [26]. Thus, the target of disease for NBS may vary based on its prevalence in a specific region. In addition, the natural history of the disease might influence the selection of the disease. With Fabry disease, an earlier study reported the prevalence of Fabry disease was similar to that of other LSDs [27]. However, growing evidences have revealed that the prevalence of the adult onset seems to be higher in some cases. For example, a mutation IVS4 + 919G > A in the *GLA* is frequently found in Taiwan; the phenotype of this mutation seems to be associated with cardiac damage with the adult onset of the disease [28]. In fact, the phenotype associated with the adult onset of Fabry disease might be rather common with different mutations in the *GLA* gene in other areas [29,30].

The analytical range is defined as the ratio of the peak area of enzyme reaction products in controls to that in the filter paper blank samples [8]. This value gives an idea for the lower detection limit of the assay. Based on previous studies, the representative values of the analytical ranges using 4 MU-mediated fluorescence assays were 4.9–16.6, but the MS/MS-based technique had larger values [8,20]. Thus, it can be presumed that the LC-MS/MS-based method offers much better quantitative results with a wider range of concentrations [3]. It is known that the patients exhibit almost no enzyme activity at the time of diagnosis. In contrast, there is a population of individuals with pseudodeficiency, the genetic variation, in which the homozygous alleles may show low enzyme activity. For example, in Japan, individuals with the G576S homozygous mutation in their GAA are part of this population. They exhibit 5–20% of enzyme activity compared to the controls with approximately 3% of prevalence [31]. It may be that every individual in this population should be identified as normal. The previous study by Liao, H.C. and ours have shown that the MS/MS-based technique offers an advantage for separating this population from that of disease-affected individuals [21,32].

In sum, an 11-plex LSD enzyme assay was examined by LC-MS/MS. The maximum interday CV% values were 10% when the activity of 11 enzymes in the rehydrated QC DBS with 100% enzyme activity was examined. The distribution of the 11 LSD enzyme activity exhibited a bell-shaped single peak. Although the number of samples was limited, the DBS for the disease-affected individuals was properly identified. Thus, future studies will be able to examine the putative cut-off values using this assay procedure. When cut-off value is to be determined, age-matched controls are to be used because leukocyte count of neonatal blood is 2–10-times higher than that of older population. The typical run-time for this assay is 2 min. This suggests that NBS for these LSD enzymes can be performed using this assay procedure.

## Appendix A Supplementary data

Supplementary material 1 Image 2

Supplementary material 2 Image 1
