# Supplementary material for: Quantification of 11 enzyme activities of lysosomal storage disorders using liquid chromatography-tandem mass spectrometry
Source: Mol Genet Metab Rep. 2018 Sep 7;17:9–15. doi: 10.1016/j.ymgmr.2018.08.005 (PMC6129719; doi:10.1016/j.ymgmr.2018.08.005)
Supplement: Supplementary file 1 — Supplementary material 1 [file mmc1.docx]

**Supplementary Figure legends**

**Supplementary Fig. 1**

Peak areas of IS for each enzyme reaction in 11-plex assay (dark gray) and 6/3/2-plex assay (light gray). Reaction products were solubilized with Reconstitution solvent (300 μL) and an aliquot (5 μL) was injected onto UPLC-MS/MS. Bar represents standard deviation (*n* = 5).

**Supplementary Fig. 2**

Calibration curves for LSD enzyme assay. Each QC DBS with 100 and 5% enzyme activity was indicated as High and Low, respectively. Solid line represents the calibration curves obtained from 11-plex assay and broken line represent the calibration curves from 6/3/2-plex assay, respectively.

**Supplementary Fig. 3**

Distribution of enzyme activity in healthy controls examined with 11-plex assay using LC-MS/MS (*n* = 200).

**Supplementary Table 1**

UPLC method for the quantification of 11-plex LSD enzyme activity in DBS using LC-MS/MS.

| LC | ACQUITY (Waters) |
| --- | --- |
| MS | Quattro Premier XE (Waters) |
| Column | ACQUITY CSH C18 (Waters) |
|  | Particle diameter: 1.7 μm |
|  | Internal diameter: 2.1 mm |
|  | Length: 30 mm |
| Column temperature | 40°C |
| Weak wash solvent | 20% acetonitrile in water |
| Strong wash solvent | acetonitrile |
| Mobile phase A | 0.2% formic acid in 5% acetonitrile/95% water |
| Mobile phase B | 0.2% formic acid in acetonitrile |
| Gradient (% B) | 0 - 0.10 min: 20% B |
|  | 0.10 - 1.00 min: 20% - 100% B |
|  | 1.00 - 1.50 min: 100% B |
|  | 1.51 - 2.00 min: 20% B |
| Flow rate | 0.6 mL/min |
| Injection volume | 5 μL |
| Sample loop volume | 10 μL |
| Autosampler temperature | 10°C |

**Supplementary Table 2**

MS instrument parameters for 11-plex LSD enzyme assay using a Waters Quattro Premier XE UHPLC-MS/MS system.

| Interface | Electrospray ionization |
| --- | --- |
| Polarity | Positive |
| Capillary voltage | 2.0 kV |
| Source temperature | 120°C |
| Desolvation temperature | 450°C |
| Flow rate of cone gas | 50 L/h |
| Flow rate of desolvation gas | 500 L/h |
| Analyzing mode | MRM |
| Dwell time | 5 ms |
| Data format | Centroid |

MRM, multiple reaction monitoring.

**Supplementary Table 3**

MRM parameters for 11-plex LSD enzyme assay using a Waters Quattro Premier XE UPLC-MS/MS system.

|  | Precursor ion  (*m/z*) | Product ion  (*m/z*) | Cone  (V) | Collision energy  (V) |
| --- | --- | --- | --- | --- |
| GAA-IS | 503.40 | 403.30 | 10 | 16 |
| GAA-P | 498.30 | 398.30 | 10 | 20 |
| GAA-S | 660.40 | 560.30 | 25 | 22 |
| GLA-IS | 489.30 | 389.30 | 22 | 12 |
| GLA-P | 484.30 | 384.20 | 22 | 12 |
| GLA-S | 646.40 | 546.30 | 23 | 19 |
| IDUA-IS | 431.30 | 322.20 | 20 | 15 |
| IDUA-P | 426.20 | 317.20 | 20 | 12 |
| IDUA-S | 602.30 | 317.20 | 35 | 14 |
| ABG-IS | 391.40 | 271.30 | 15 | 12 |
| ABG-P | 384.40 | 264.30 | 15 | 12 |
| ABG-S | 546.40 | 264.20 | 20 | 15 |
| ASM-IS | 405.40 | 264.30 | 15 | 20 |
| ASM-P | 398.40 | 264.30 | 15 | 20 |
| ASM-S | 563.40 | 184.00 | 15 | 30 |
| GALC-IS | 417.40 | 264.30 | 20 | 18 |
| GALC-P | 412.40 | 264.30 | 20 | 15 |
| GALC-S | 574.50 | 264.30 | 20 | 25 |
| ID2S-IS | 649.30 | 364.30 | 25 | 20 |
| ID2S-P | 644.25 | 359.30 | 25 | 20 |
| ID2S-S | 724.30 | 359.23 | 26 | 20 |
| NAGLU-IS | 423.20 | 314.20 | 25 | 20 |
| NAGLU-P | 420.20 | 311.20 | 25 | 20 |
| NAGLU-S | 623.40 | 311.30 | 25 | 20 |
| GALN-IS | 690.40 | 378.30 | 35 | 25 |
| GALN-P | 685.40 | 373.30 | 25 | 20 |
| GALN-S | 765.30 | 373.30 | 15 | 20 |
| ARSB-IS | 662.40 | 350.30 | 25 | 25 |
| ARSB-P | 657.35 | 345.20 | 25 | 20 |
| ARSB-S | 737.30 | 345.20 | 15 | 23 |
| GUSB-IS | 441.30 | 332.30 | 35 | 15 |
| GUSB-P | 434.30 | 325.30 | 35 | 15 |
| GUSB-S | 610.70 | 325.40 | 35 | 15 |

IS, internal standard.
