# Supplementary material for: Quantification of 11 enzyme activities of lysosomal storage disorders using liquid chromatography-tandem mass spectrometry
Source: Mol Genet Metab Rep. 2018 Sep 7;17:9–15. doi: 10.1016/j.ymgmr.2018.08.005 (PMC6129719; doi:10.1016/j.ymgmr.2018.08.005)
Supplement: Supplementary file 2 — Supplementary material 2 [file mmc2.pdf]

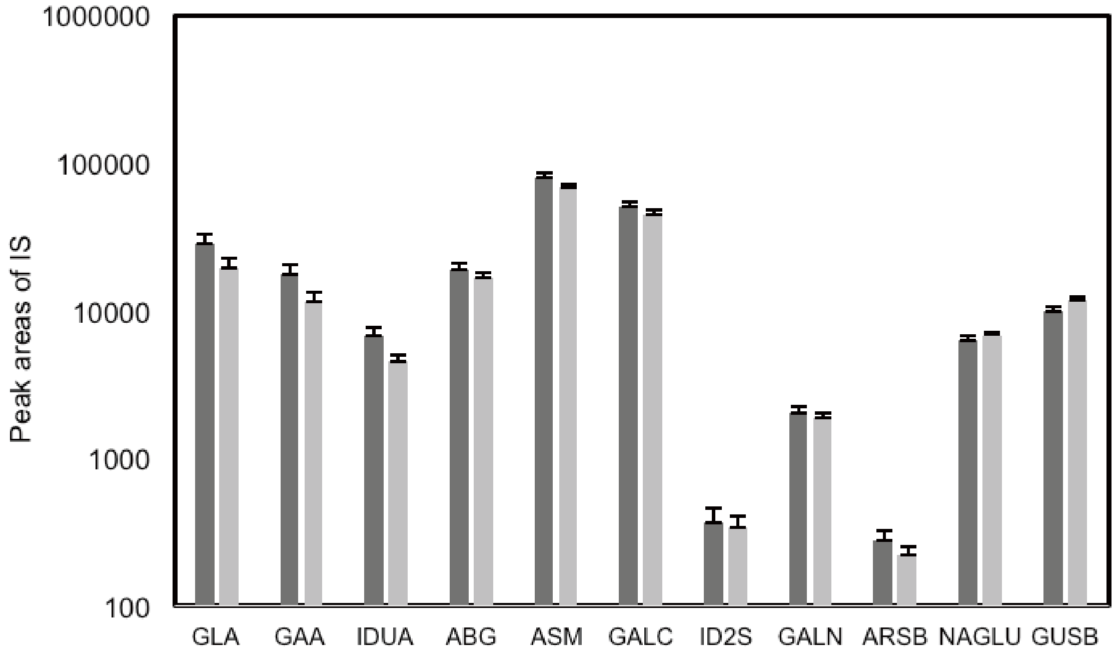

Supplementary Fig. 1
Ohira M et al.

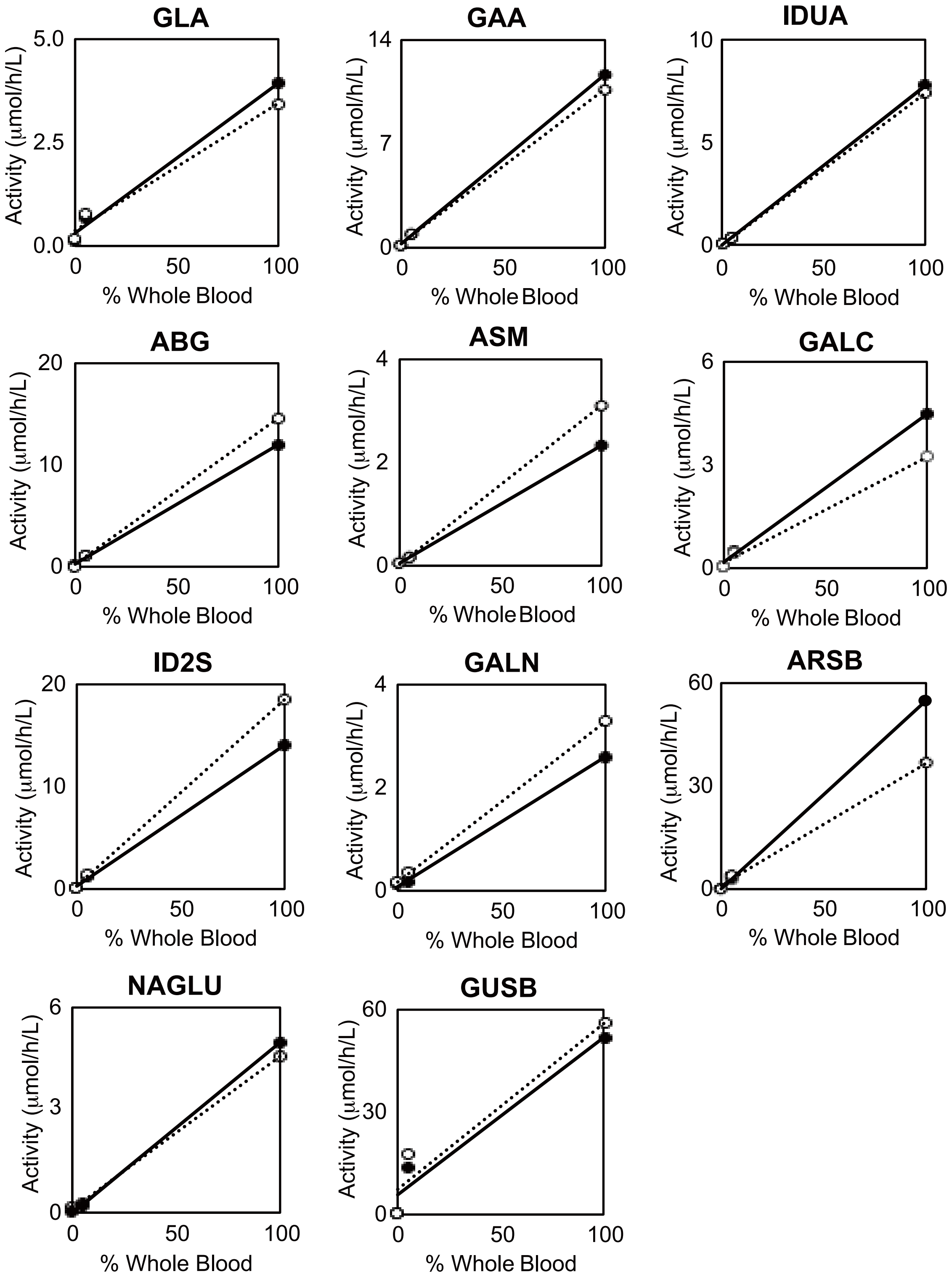

Supplementary Fig. 2
Ohira M et al.

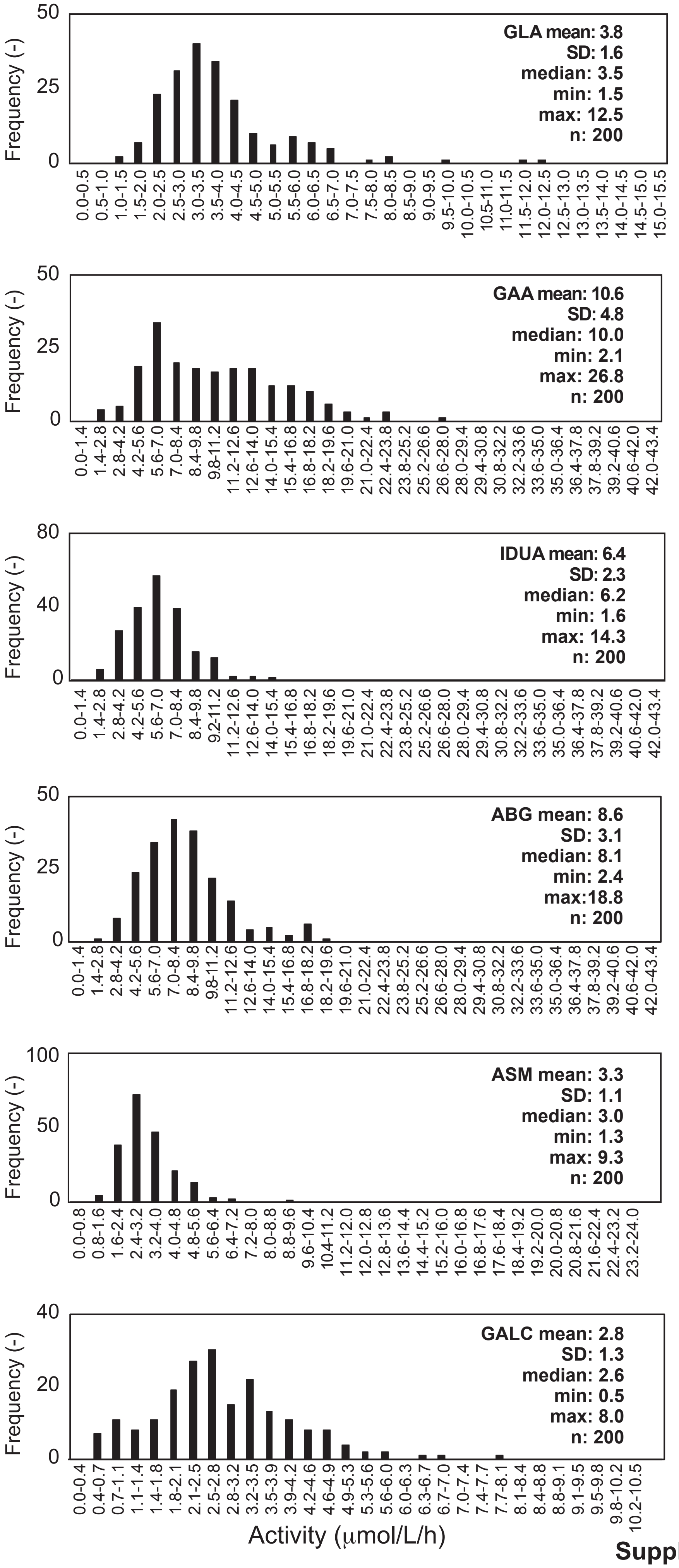

Supplementary Fig. 3a
Ohira M et al.

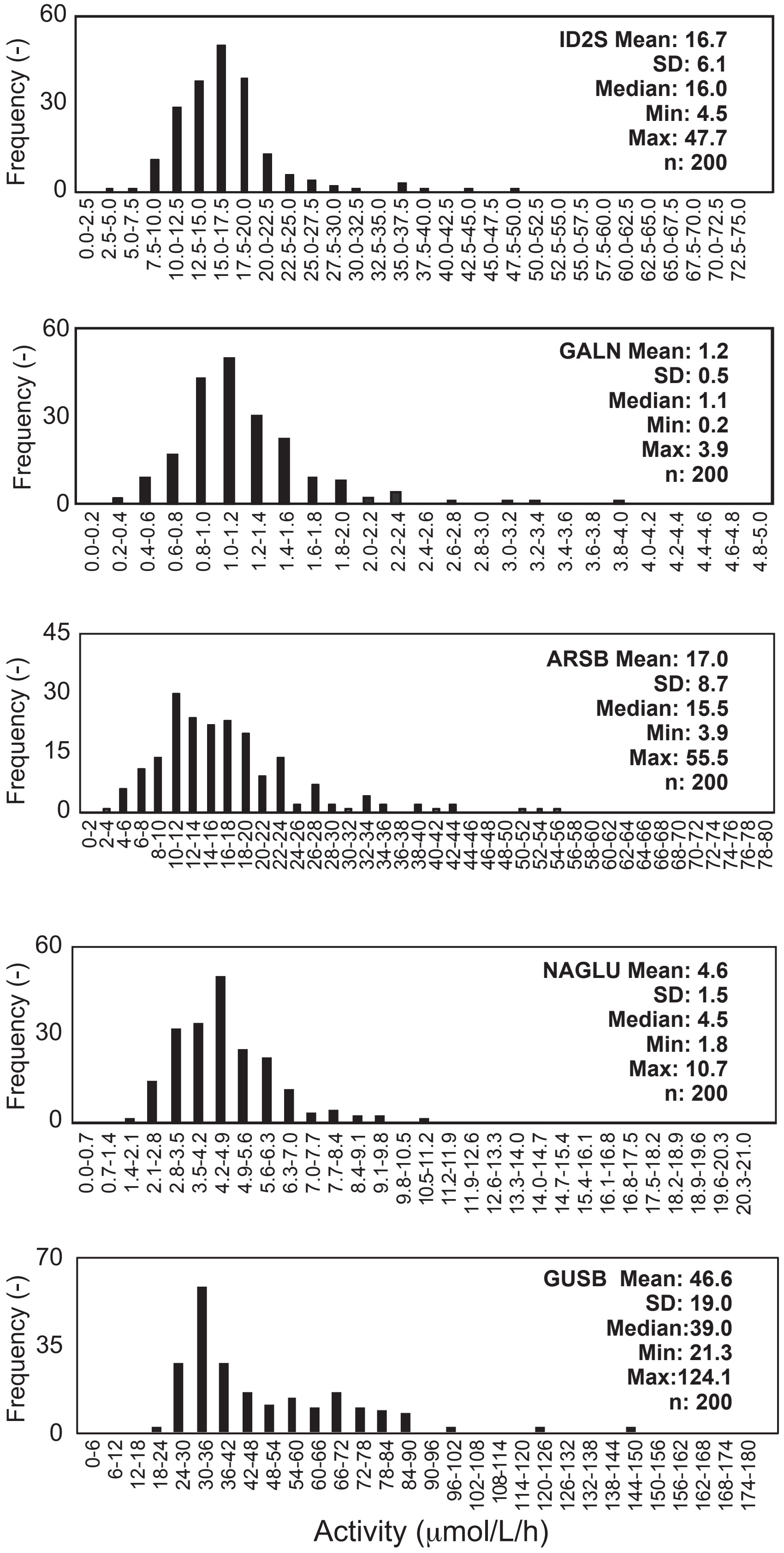

Supplementary Fig. 3b
Ohira M et al.
